# “Stay indoors with *Purdah,* men will make the money”: A qualitative study investigating women’s microfinance participation and mobility practices in Bangladesh

**DOI:** 10.1371/journal.pone.0346323

**Published:** 2026-04-02

**Authors:** Kanig Fatema Akter Bristi, Tunvir Ahamed Shohel, Taufiq-E-Ahmed Shovo, Maherun Nahar Mumu, Hamalna Nizam

**Affiliations:** 1 Sociology Discipline, Social Science School, Khulna University, Khulna, Bangladesh; 2 Development Studies Discipline, Social Science School, Khulna University, Khulna, Bangladesh; 3 English Discipline, Arts and Humanities School, Khulna University, Khulna, Bangladesh; Duke University, UNITED STATES OF AMERICA

## Abstract

**Purpose of this paper:**

This study aimed to investigate the impact of *Purdah*, a cultural norm, on the economic independence of women who have received microfinance in Bangladesh. The study also sought to understand whether the *Purdah* norm has an influence on microfinance loans, such as use, control, and repayment, relevant to female borrowers’ mobility practice. Perception, cultural value, and *Purdah’s* implications for everyday life were also considered.

**Design/methodology/approach:**

We selected women’s groups in development programs, such as microfinance, as the case for this study. By using a snowball sampling technique, we identified 25 microfinance recipients for interview– mostly women and their spouses living in the Dumuria and Tala Upazilas in the Khulna division of Bangladesh. The research employed thematic analysis to draw conclusions and explain the findings.

**Findings:**

The study findings show that traditional norms associated with *Purdah* hinder women’s economic engagement and that the practice of *Purdah* among women is institutionalized from a very young age, primarily before marriage. The findings further explain that *Purdah* norms limit women’s mobility, act as a cultural barrier to IGAs, create economic dependence on men, and enable men to control microfinance loans. Therefore, women are not fully in control of their microfinance loans, and microfinance participation appears to be a less satisfactory form of financial inclusion for participants.

**Practical implications and value of paper:**

This study suggests that viewing *Purdah* solely as a religious requirement limits understanding of its cultural roots. Cultural interpretation is needed to shape policies and reduce Purdah’s impact on women’s mobility.

## Introduction

*Purdah* is a Hindi-Urdu word that means covering the whole body with a veil. It is usually practiced by Muslim women, but is also a familiar practice among other religious groups in our study location, as it is a cultural norm there. In this study, we used *Purdah* to refer to not only the religious practice but also the cultural norm that was in operation among the participants. *Purdah*is a religious social exclusionary practice that not only governs women’s seclusion at home but also enforces their exclusion from public settings and constructs specific gender identities [1,2]. *Purdah* has also become a cultural norm that is practiced and understood beyond religious seclusion, creating political agency, providing certain rules and restrictions for women towards mobility and communication. Although *Purdah* has its roots in religion, this study investigated *Purdah* as a cultural norm beyond its religious context. Interpretations of *Purdah* instruct women to conceal their appearance, avoid making eye contact with men, and steer clear of crowded areas, such as public places, community spaces such as supermarkets, streets, and parks [1,3–8]. The cultural practice of *Purdah* norm imposes constraints on women’s mobility, which are mandated by traditional conservative practices that discourage women from directly engaging in economically productive activities, especially those that take place outside the threshold of one’s home [5,9,10].

In Bangladeshi culture, the practice of *Purdah* restricts women’s mobility and prevents them from taking part in many spheres of society, including the workforce and higher education; instead, they are allowed to participate in socially accepted semi-private areas, a negotiation they do in their regular everyday experiences [3–5,7,11–13]. The restrictions imposed by *Purdah* prevent women from freely pursuing opportunities for various advancements, such as education and economic independence [4,14]. Further, contact or communication with men beyond kinship or family ties is seen as dishonorable and immoral; hence, women are generally kept isolated and restricted to their houses [4,14].

Microfinance, meanwhile, is well known as a development strategy for increasing women’s opportunities, including mobility, income, and decision-making [15]. The Millennium Development Goals (MDGs) for gender equality and women’s empowerment recognize microfinance as an important instrument for advancing women’s lives [16]. The use of microfinance by women is said to increase their mobility and presence in public spaces such as marketplaces [17,18]. Microfinance is believed to increase women’s mobility, ability to make purchases and important household decisions, ownership of productive assets, legal and political understanding, and engagement in public campaigns and protests [19,20]. Evidence claims that female microfinance borrowers experience higher satisfaction with their financial security and accomplishments [21].

However, restrictions on women’s mobility in Bangladesh, as the World Bank’s recent study notes, contribute to the country’s low female employment rates [22]. There is evidence that women’s involvement in the labor force and income generation is minimal, and patriarchy (e.g., *Purdah*) remains a fundamental barrier to either female-led production processes or women’s participation in the labour market [2–5,7,12,13,23]. For example, mobility restrictions due to *Purdah* prevent women from engaging in financial management [4,24,25]. Nevertheless, advocates argue that improving women’s condition through economic emancipation, such as Income Generating Activities (IGAs) or entrepreneurship, can enhance women’s economic engagement [19,26–28]. Though, as many researchers suggest [4,29,30], women’s mobility may have increased through microfinance, the *Purdah* norm also has significant control over women’s lives, which raises the question: how far or to what extent has social change (e.g., mobility emancipation) impacted the *Purdah* norm or vice versa?

Therefore, from sociological viewpoints, research is needed to understand the *Purdah* norm within the cultural compound; in particular, it is necessary to investigate women who are microfinance beneficiaries and are perceived to enjoy greater mobility in patriarchal settings such as Bangladesh [19,31]. The supporting evidence [19,31] from microfinance programs and Microfinance Institutions (MFIs) shows that microfinance beneficiaries have greater mobility access due to their microfinance participation. Therefore, this study aims to: a) investigate the impact of *Purdah* norms on the economic independence of women who have received microfinance in Bangladesh; b) understand whether the *Purdah* norm influences factors of microfinance loans such as their use, control, and repayment relevance to female borrowers’ mobility practices; and c) comprehend *Purdah’s* perception and cultural value, as well as its implications for everyday life. To achieve this, 25 microfinance beneficiaries from Bangladesh were interviewed as data sources. A qualitative methodological approach was incorporated, and a semi-structured in-depth interview (IDI) guide was used for data collection.

We believe that pursuing this research will contribute to microfinance literature by addressing the gap between cultural norms around women’s mobility and their impact on economic autonomy. As women’s economic autonomy and participation require their freedom of movement, many success stories have evidenced that women’s microfinance participation challenges their restricted mobility and increases their public participation [17–20]. However, over time, many researchers [,^5^,^24^,^25^,^31^,^32^] have documented that women’s participation in microfinance loans merely creates the illusion of their greater mobility within their communities. We examine the *Purdah* norm as a cultural barrier affecting women’s mobility and economic independence, addressing a gap in existing research. This study intends to explore *Purdah*, microfinance use, and women’s autonomy, aiming to contribute uniquely to microfinance literature.

## Literature review

### *Purdah* norm and women’s mobility restrictions

*Purdah* is primarily observed by Muslims and is often used to define cultural behaviors or organizations [33]. From a wider perspective, *Purdah* is adherence to a cultural or religious expectation (e.g., Muslim) that is expected of follower communities, such as Muslim women wearing hijab, which is considered modest attire. However, *hijab* is also used as fashion clothing in some areas, irrespective of religious norms [34]. In Bangladesh, *Purdah*-practicing women are expected to wear a particular garment called the *Burqa*, an overlay conceals the entire body, including the face [14]. However, the *Purdah* norm may not limit modest wear and expected behavior to clothing alone. In Bangladesh, girls are taught to respect and follow cultural norms such as *Purdah* from a young age [5]. *Purdah* influences and shapes women’s decisions about their responsibilities; it remains a dominant factor in their lives [1].

In Bangladesh, women are expected not to engage in direct productive endeavors, especially outside the home, due to the need for *Purdah* regulations [10,20]. Accordingly, women’s mobility is limited to gendered spaces [35] and cultural norms like *Purdah* limit access to services and create difficulties in traveling long distances [36]. In addition, women practicing *Purdah* must control their behavior in certain ways to preserve their identities and moral standings compared to women who do not adhere to *Purdah*. The latter are free to go where they wish and work where they want; consequently, they are more likely to feel empowered [20,37]. In Bangladesh, where a predominantly patriarchal Muslim culture prevails, *Purdah* plays a significant role in shaping women’s lives, as they must adhere to its rules in the country’s male-dominated public sphere [38].

### Patriarchy and women’s subordination

Patriarchy is a system of social structures and practices in which men rule, oppress, and exploit women [39]. In a patriarchal society, men are conditioned to hold all positions of power and benefit economically at the expense of women [40]. According to Sesay and Odebiyi [41], patriarchy gives men the material means to dominate women, since it elevates the former to a position of power over the latter [41]. Thus, culturally controlled gender education has long-lasting effects on both men’s and women’s agency [42]. According to Kate Millett’s theory on women’s subordination, women are a dependent sex class under a patriarchal power structure [43]. Since men are socialized to see women as fundamentally different from themselves, they treat them as second-class citizens in society [44]. Women homemakers are seen as economically reliant on their husbands despite their jobs being physically demanding, monotonous, and never-ending [45]. Several studies [3,5] have shown that, in patriarchy, women’s subjugation is founded long before their marriage (i.e., from their childhood); they are perceived as not being an asset like men are, and, after marriage, are considered a burden to men’s economy as they are financially dependent on their husbands. Both patriarchy and women’s acceptance of patriarchal norms contribute to the oppression of women [46]. In this context, Jani and Parekh, [47] have argued that *Purdah* is a patriarchal norm that exists in Muslim culture and serves to oppress and control women throughout their lives via rituals and customs that provide them with a false sense of propriety and safety. Its regimental use is intentional; it serves the self-interest of a specific group of people.

## Theoretical framework

We used two theories, gender role construction theory and moral obligation theory, as our theoretical framework. Through this, we argue that culture is a supreme force that constructs gender-specific roles and obligations in practice based on women’s mobility norms. Culture-based gender-specific mobility norms have developed over generations as a moral obligation for women, impacting women’s gender-specific power relations, entrepreneurship, IGAs, and financial control.

### Culture and gender: Construction of women’s role

The first theory used in this study was gender role construction; we adopted this to understand how women’s gender roles are culturally constructed and practiced. In this section, we argue that the construction of women’s roles has a cultural foundation; by practicing their culture-taught gender roles over generations, women have normalized their subordinate roles in an innate fashion compared to men. We used gender role construction theory to understand *Purdah* norms’ perception(s) and practices through the development of gendered identities. It is worth mentioning that the proposition of this theory also grounded our second theoretical proposition, “the moral obligation of *Purdah* and economic restrictions for women”. The gender role construction theory has a long history [48] of evidence from C. H. Cooley and G. H. Mead to modern thinkers such as Connell, John *et al.,* [48] and Gauvain & Perez [49]. C. H. Cooley and G. H. Mead considered as symbolic interactionists, studied at gender socialization theory in the early 20^th^ century and theorized that children’s socialization process explains how men and women develop their social identities and roles.

To liberate the century past contribution of C. H. Cooley and G. H. Mead [48], and to reconnect our concepts to the present society, we conceptualize gender role construction by blending two theories: interactionism, which considers gender identities as the consequence of frequent social interactions, and structuralism, which explains how gender identity develops within social systems [48]. Our theoretical paradigm for explaining gender learning especially emphasizes developing sociological knowledge. It is observed that family members, especially parents, significantly impact young children by instructing them about appropriate domestic conduct and distributing work according to gender roles [48]. Raising and caring for children, giving toys, selecting sports, and modelling gender-stereotypical divisions of work at home are all activities that contribute to gender socialization [49]. Described by Connell in 2005 as the “patriarchal dividend” [50], men in patriarchal societies are socialized to have more masculine (i.e., desirable) characteristics than women. Such disproportionate mobilizing norms have also been found to moderate both men’s and women’s roles; in culture, women’s lack of mobility may be expounded by cultural features [2,3,6].

### Moral obligation of *Purdah* and economic restrictions for women

The second theory we incorporated in our theoretical framework was moral obligation; we did so in order to understand *Purdah* as a culturally driven obstruction for women’s economic participation. The second theoretical proposition was founded upon an analytical frame that argues that *Purdah* is a moral obligation for women. Moral obligation is a normative feature in life that comprehends an individual’s moral duty to do or follow something commonly practiced in a particular society [51]. In patriarchy, the gendering process is institutionalized by norms, order, and practices, whereby girls, from childhood, are obliged to avoid mobility beyond the home, avoid greeting or meeting unknown people, and cover their bodies with clothes as prescribed by their elders, families, or community [6]. These obligations are not only institutionalized but also accompanied by deeper cultural attributes. The moral obligation of *Purdah* for women also involves the imposition of societal constraints (e.g., confinement at home, home-based division of labor) and eventually lessens their economic opportunities [2].

Moreover, the moral stance of *Purdah* is to curb women’s free mobility by establishing institutions such as marriage and making men the decision-makers of the family and economic regulations [52]. No matter the difficulties (e.g., domestic violence, economic dependency on men) women faces, their moral obligations restrict their mobility beyond the house for any purposes such as economic dealings or employment [3,52]. When embodying the moral obligation of the *Purdah* norm, economic dealings (e.g., entrepreneurship, investment, purchase, uses, earning) are culturally absent from women’s roles; it has become normative for them to rely on their male family members for livelihood [4,5].

We conceptualized our analytical framework within our theoretical context; the intent was to examine Purdah and its impact on economic independence for women who receive microfinance loans for entrepreneurial activities. We emphasize that it is necessary to have a firm grasp on the gender role construction process, as discrimination based on gender is mostly a cultural issue. In particular, girls are taught from infancy that they are inferior to their male counterparts in intelligence and potential opportunities [2,4–6]. In various aspects, the incompetence visualized in girls is nurtured and normalized as a moral obligation by culture [3]. To better comprehend the conceptual construction of gender power relations in society and the resulting limits on women’s mobility, the gender role construction and moral obligation theories are useful, as they help us understand patriarchy and its culture-born features.

## Gaps in literature

Many prior studies have documented that microfinance participation has improved women’s income generation, consumption, and household expenditure [19,26–28,53]; overall, it has helped women escape poverty by offering economic emancipation [54]. However, other researchers have found that women’s participation in microfinance rarely improves their quality of life or their empowerment [4,5,24,25,55,56]. Among these findings, two common areas are crucial: first, women in microfinance handed over their money to men [4,5,31,57]; and second, no significant improvement in women’s mobility restrictions was observed [4,5,24,58]. Researchers such as Ahmed [55] have argued that in patriarchy, women’s role as ideal wives (i.e., burdened with care work and mobility restrictions) and men’s masculine dominance decrease women’s control over finances through culture and practice. However, to our knowledge, there is a shortage of literature that critically examines *Purdah’s* impact (both direct/indirect) on microfinance-receiving women in Bangladesh. From this perspective, this research intends to fill the literature gap by looking at the *Purdah* norm, a concept of culture, to understand women’s financial behavior (e.g., entrepreneurship, IGAs, financial control) in Bangladesh.

### Methodology

#### Research design and method.

A qualitative strategy was used for this research, as it was considered the best option for learning more about the topic from the perspective of microfinance recipients [59]. Accordingly, to investigate women’s mobility restrictions associated with the *Purdah* norm, we used a qualitative approach to comprehend the objective bases for our subjective experiences, judgments, and views, while accounting for human behavior, thinking, and action [60]. We used the In-Depth Interview (IDI) method to collect data, which not only allowed us to elicit respondents’ subjective experiences regarding the *Purdah* norm but also enabled us to observe their emotional reactions to the interview. During the data collection period, experiencing potential discomfort and managing participants’ emotional stages are essential for the researchers [61]. In a situation like that, we did our best to alleviate any potential discomfort (e.g., immediately stopping IDI, consoling her, asking if we may reschedule the interview if she is not comfortable, and informing her about the support system available from the local government) that our research participant may have felt as a result of taking part in this study.

#### Study subject.

This study’s respondent population included women and their husbands who had received microloans while adhering to the traditional practice of *Purdah* in the *Dumuria* and *Tala Upazila*s [see S1 Map – which was generated by the authors of this paper using ArcGIS (version 10.7.1) solely for this current research.] in the Khulna division. Both *Dumuria* and *Tala* Upazilas were purposively considered for being mainly rural areas respecting the classical gender norms, especially *Purdah*. Our study was more focused on the context of these norms within a rural microfinance environment where mobility restrictions are maximized. We selected study participants based on specific criteria, including regular microfinance borrowers [who regularly participates in micro-finance programs was defined to include borrowers that were active in their participation of the micro-finance program, i.e., engaged in loan cycles on an ongoing basis; made timely repayments to the micro-finance organization; and regularly attended weekly or monthly micro-finance group meetings. The level of borrower participation was determined based on the participant’s self-report, and when possible, through documentation maintained by micro-finance field officers or group leaders.] with at least 5 years of experience, who agreed to take part in the interview, and who had strong adherence to the *Purdah* norm. In this research, participants’ adherence to *Purdah* norms was defined by their observable socio-cultural and mobility-related behaviors. A participant’s adherence to *Purdah* was defined by her reporting that she maintained gender-segregated interactions within the home; that she limited her movements outside of the home unless accompanied by a male guardian or family member; and that she wore culturally acceptable veiling practices (e.g., hijab, niqab, etc.) while in public spaces. These measures of adherence to *Purdah* were initially collected via self-report behavioral descriptions as part of the initial screening process and subsequently verified through community gatekeepers and micro-finance group facilitators who had prior knowledge of the participants’ behaviors.

We also included microfinance participants who have been local to our study area for at least 5 years. We excluded women who are irregular participants in microfinance programs such as stopped using microfinance, infrequently attended group meetings, or had a borrowing history for less than 5 years, as well as women whose Purdah practices were either flexible or unrestrictive and therefore did not limit their mobility, were all removed in order to allow for consistency with the studies’ research design that focused on women’s restricted mobility within social and cultural systems. For decades, Bangladesh has been firmly rooted in patriarchy; the rural areas are the stronghold of cultural discrimination against women, who have limited or no mobility there due to a strong tradition of *Purdah* [2–8,12,13]. Our assumption was that study participants would have a close connection to the *Purdah* norm and its everyday practices. The *Purdah* norm limits women’s decisions to leave their homes without the permission of men (e.g., husbands, fathers, brothers). Women rarely travel outside the house, usually with the household head’s permission and accompanied by men or family members to visit the MFIs’ offices.

Using the snowball sampling method, 25 microfinance beneficiaries, all of whom were female microfinance borrowers or their husbands (see Table 1), were selected as the research participants. At the same time, it is assumed that exercising the *Purdah* norm is a barrier to women’s mobility. In this context, it is important to note that the study’s target sample was intentionally focused on Muslim women, the main population of interest, given the significance of *Purdah* norms in their daily lives. A lesser number of Hindu participants and men (Husbands) were included with an eye for drawing comparative and contextual insights to complement the study.. The reason is that in rural Bangladesh, *Purdah* is not only practiced as an Islamic religious belief but also often acts as a wider cultural norm. Among Hindu communities, especially in rural settings and particularly in our study location, *Purdah-type* practices are varied and may mean acts of modesty and respect toward elders rather than actual prescriptions dictated by religion. Thus, by including Hindu participants, we could encapsulate all these finer side notes and highlight that from the perspective of rural cultural life in general, restrictions on women’s mobility do not take on an Islamic viewpoint alone. Their inputs helped to triangulate the Muslim participants’ responses, revealing how *Purdah* is understood beyond religion and thereby confirming the interpretation of *Purdah* as a cultural and patriarchal agency cutting across religious lines. Therefore, religious and gender dilution are consequences of the targeted nature of our research question, rather than a conscious attempt to achieve demographic balance.

**Table 1:** Personal Information of the Participants.

Participants	Type of Participants	Age	Religion	Family Decision Maker
Informant-1	Female Borrower	25	Islam	Husband
Informant-2	Female Borrower	55	Islam	Daughter
Informant-3	Female Borrower	43	Islam	Husband
Informant-4	Female Borrower	55	Islam	Both Husband and Self
Informant-5	Female Borrower	51	Islam	Husband
Informant-6	Borrower’s Husband	38	Hinduism	Husband
Informant-7	Female Borrower	40	Islam	Husband
Informant-8	Borrower’s Husband	46	Hinduism	Husband
Informant-9	Female Borrower	35	Hinduism	Husband
Informant-10	Female Borrower	45	Hinduism	Husband
Informant-11	Female Borrower	40	Islam	Son
Informant-12	Female Borrower	30	Islam	Husband
Informant-13	Female Borrower	45	Islam	Son
Informant-14	Female Borrower	30	Islam	Husband
Informant-15	Female Borrower	25	Islam	Husband
Informant-16	Female Borrower	22	Islam	Husband
Informant-17	Female Borrower	35	Islam	Husband
Informant-18	Female Borrower	60	Islam	Husband
Informant-19	Female Borrower	38	Islam	Self
Informant-20	Female Borrower	35	Islam	Husband
Informant-21	Female Borrower	35	Islam	Husband
Informant-22	Female Borrower	50	Islam	Self
Informant-23	Female Borrower	30	Islam	Husband
Informant-24	Borrower’s Husband	35	Islam	Husband
Informant-25	Female Borrower	20	Islam	Husband and Father-in-law

(Source: Field investigation).

#### Background information on research participants.

The following table (see Table 2) illustrates the background information on the research participants. Most participants (N = 13) were between 35 and 49 years old and identified as Muslim Most participants (N = 21)., Husbands were found as the primary breadwinners in most households (N = 18). Most participants (N = 18) had a family income of BDT 6000–15000 per month, and a majority (N = 15) of the earning members of the family engaged in daily labor.

**Table 2:** Participants’ Background Information.

Personal information of the participants	Class interval	Number of participants (N)
Age (in years)	20-34 35-49 50-64	7 13 5
Gender	Male Female	3 22
Family size (in members)	1-5 6-10	20 5
The monthly income of the family [in Bangladeshi Taka (BDT)]	6000-15000 16000-25000	18 7
Religion	Islam Hinduism	21 4
Head of the family	Solely Husband others	18 7
Occupation of the earning member	Agriculture Daily labor Other sectors	9 15 1

(Source: Field investigation).

#### Interview outline.

Based on a study of existing literature, we developed a semi-structured in-depth interview guide to collect first-hand accounts. The semi-structured in-depth interview guide included questions about Purdah’s impact on female microfinance recipients’ mobility, as this approach yields a higher response rate than other data collection methods. Nonetheless, trust was fostered through a manageable sample size and culturally sensitive strategies, such as using same-gender interviewers, conducting interviews at locations selected by participants, and assuring strict confidentiality. These efforts increased the likelihood that researchers obtained complete data [62].

#### Data collection protocol.

The research data were gathered in two phases. In the first phase, 13 participants were interviewed in July 2022 in Dumuria *Upazila* of Khulna Division. Phase 2 consisted of 12 in-depth interviews conducted in August 2022 in Tala *Upazila* of Khulna division. Each interviews lasted about 40–50 minutes on average. Questions were asked in Bengali during the interview session, and the interviews were recorded verbatim with the participants’ permission. All interviews were treated as confidential, and participants were free to withdraw at any time.

The researchers did not coerce anybody into participating in the study. Only participants who volunteered willingly to provide information were considered. The researchers guaranteed that the participants would remain anonymous throughout the research process. Accordingly, in line with the *Purdah* practice, interviews with women were conducted either at their homes or in a place agreed by both parties, where the women felt comfortable and at liberty to talk. Male family members were not present during interviews, although, in rare instances, a female family member may have stood nearby at the participant’s wish. Apart from this, we need to mention that to show respect for the prevailing culture and gender norms, female researchers interviewed female participants and male researchers interviewed male participants. This ensured that Purdah was respected and that trust and openness were encouraged during the interviews.

#### Data analysis.

We followed the guiding principles suggested by Ritchie and Lewis [63] and Braun and Clarke [64] for developing a thematic analysis. The guide to developing thematic analysis suggested that it required a familiarizing phase of the data relevant to our research aim, generating initial codes from the transcribed/translated data set, grouping the codes under potential themes, and then running inclusion and exclusion techniques (e.g., reviewing and revising codes) to generate the final narrative for research.

Following the above process, after the interviews were finished, audio recordings of the interviews were transcribed into *Bengali* text format and then translated into English by the researchers. For this, the researchers followed the back-translation process in order to maintain the translation’s accuracy and consistency. Subsequently, NVivo 12, a program for managing and processing qualitative data, was used to code and interpret the transcribed data into themes. Each contributor helped to summarize key ideas and set the scene for the topics. The data was evaluated using thematic analysis because this allows researchers to identify the core themes that best explain their research phenomenon. However, it is essential to clarify that once the answers developed into repetitive patterns and began contributing only trivial new themes, mainly from the Muslim females, who were at the center of the study, saturation was considered to have been attained.

The findings from the thematic analysis highlighted the most salient meaning groups within the dataset. By comparing how often each topic appeared, we could connect the current study’s findings and the real world. In addition, after each interview, we discussed and agreed on the data to include in the first report. Meanwhile, we thoroughly analyzed the interview data to identify and explain any anomalies or rule them out entirely. To complement the emic and etic perspectives of the data, the authors avoided personal bias; this maintained the study’s objectivity. Comprehensive insider (i.e., participants’ viewpoint) and outsider (i.e., theoretical understanding with wider viewpoint) perspectives were considered while collecting, processing and analyzing the data.

**Fig 1:**
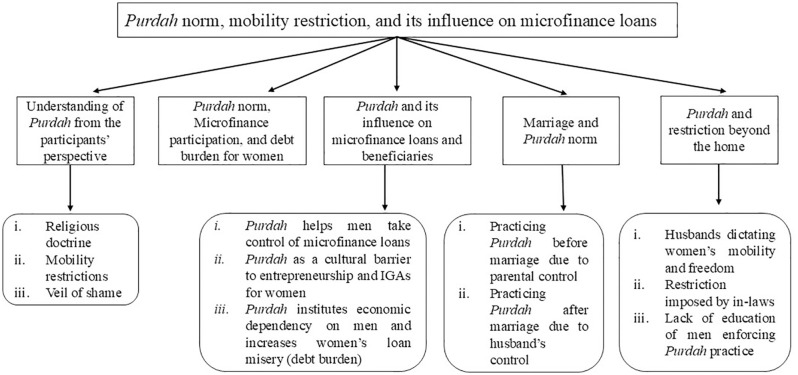
Themes conceptualizing the aim/objective of this research. Five themes were identified from our data to explain the study findings, with four of these themes comprising several sub-themes. a) The first theme conceptualizes the understanding of *purdah;* b) the second theme shows how *purdah norms* escalate debt burden for female loanees; c) the third theme contains the impact of *purdah* on microfinance beneficiaries by letting men take control over female loans; d) the fourth theme left evidence on *purdah* practice as a normative practice for women before and after marriage; e) the fifth theme showed how *purdah* placed as a normative restriction against women’s mobility beyond home**.**

The major themes developed from the field data to achieve the aim/objective of this study were conceptualized in a framework (see Fig 1 – used in this research was created by the authors of this paper specifically for this study.).

#### Ethical issues.

The ethical clearance committee of Khulna University accepted this research (Protocol No. KUECC-2022/06/17). All participants gave informed consent before the interviews. Each author also confirmed that no unethical practices, like data falsification, were involved in this research.

## Findings

The findings section presents the key qualitative data, organized into five themes. The first describes perceptions, cultural values, and implications of *Purdah* in daily life, and then its effect on microfinance participation. Next, it examines the impact of *Purdah* norms on the economic independence of women who received microfinance.

1 **Understanding of *Purdah* from the participants’ perspective**

25 study participants were interviewed about their understanding of what *Purdah* entails; the majority agreed that it included dressing modestly, not leaving the home, and listening to and obeying one’s spouse and the rule of almighty *Allah*. However, this investigation revealed three distinct perspectives relating to the extent to which the participants grasped the meaning of *Purdah*.

1.1 Religious doctrine

Seven participants (out of 25) said that Muslim women wear the veil as part of *Purdah* out of fear of Allah and to follow the religious lifestyle shown by the Prophet (SM) (PBUH) Muhammad (SM), (PBUH), was a religious leader and the last prophet who was called as the founder of Islam. Muslims from all backgrounds strive to follow his example since he was the designated recipient of the divine revelations and the messenger from Allah. From his birth in Mecca in the year 570 CE until his death in Medina in the year 632, Muhammad lived his whole life in what is now Saudi Arabia. The female participants said that they adhered to *Purdah* norms in order to appease their superiors (i.e., husbands, fathers, and in-laws). They insisted that Muslim women must observe *Purdah* so that they could enjoy eternal bliss in paradise. Participant #15, a woman with five years of experience in microfinance programs, gave the following statement:

“By Purdah, I mean the order of Allah and the lifestyle shown by our Prophet (SM). If I maintain Purdah properly, people will appreciate me, and it will protect me from “Kunazar”, (a Bengali word that means “evil eye”. Here, this word is used to indicate that Purdah is a protector from the evil eye. It refers to wishing someone bad luck or illness, which is generated through jealousy.).My husband will also be happy. As a woman, I must make my husband happy. If I do it properly, Allah will be happy with my deeds” (Participant #15, age 25).

Meanwhile, participant #14 said that following *Purdah* must be followed by wearing the *Khas* veil with no hesitation. *Khas veil* means following *Purdah* very strictly. Bengalis use this word to express their *Purdah* norm in a very conservative way. *Khas veil* refers to women who consider following *Purdah* norms to be their main motive in life; they cover their whole body with a veil, totally avoid going outside, and lead a restricted life out of fear of Allah. By this, she meant that one must be morally and intellectually pure in order to adhere to Islam’s strict guidelines. She made a case for the practice of *Purdah* from a moral and religious standpoint. Having worked in the microfinance industry for six years Participant #14 claimed that:

“By Purdah, I mean that Muslim women must follow it. Mother Fatima used to follow Purdah. It is the order of our last Prophet from Allah. However, I also think it is possible to satisfy Allah by following prayer, being honest, and practicing Khas Purdah. Practicing Khas Purdah without being honest and religious is useless” (Participant #14, age 30).

1.2 Mobility restrictions

Among the 25 participants interviewed, 10 identified *Purdah* as a cultural norm that prevents women from “going outside” the home, whereas men are not subject to the same restrictions. By “going outside”, the participants were referring to limitations on women’s mobility beyond the home, which is not the case for men. For example, (mostly adult) men can leave their household at any time and for any reason without seeking permission from anyone. These movements beyond home might be for IGAs, emergencies (e.g., health issues, purchasing goods), or casual purposes (e.g., roaming around, visiting shops or markets, or sports). Women, however, understand culturally they lack the mobility enjoyed by men and are instead confined to the home. Even for a short visit to a neighbor’s house, a woman needs to seek permission from the household head (generally a man) or inform them before or after the visit. Therefore, the limitation is also an obligation for women to comply, since it originates with the home’s decision-maker (i.e., a husband or father). Participant #23 (a woman, from a dual insider perspective, who had worked in the microfinance industry for seven years as well as a borrower) explained this as follows:

“By ‘Purdah’, I mean that you cannot go in front of a man; you must speak in a lower voice, stay indoors even within the home, obey your husband’s order, and pray five times a day” (Participant # 23, age 30).

Observing *Purdah* is substantially linked to mobility restrictions and concealing one’s body from others’ sight, as explained by participant #17, a woman who had been involved in microfinance for eight years. She further explained that, as women mature, they may be forbidden from greeting male members of their own families:

“By the concept of Purdah, I understand [it as] not only staying inside the home but also covering my face in front of my husband when we meet or greet, being polite with him and obeying his will” (Participant #17, age 35).

1.3 Veil of shame

13 of our interviewees perceived *Purdah* as including the wearing of a veil covering one’s head and body. It requires full-body coverage, including gloves and socks. This practice is widely recognized throughout cultures as a symbol of respect for women. Adult women, according to the participants, are discouraged from speaking to their fathers unless properly clothed and have a veil over their faces. Participant #24, an older married man whose household had benefited from microfinance for 12 years, reported that:

“I understand, Purdah, that as a woman, she must not show her face to outsiders. If required, she must not go in front of other men without my permission and without covering her face. She is only allowed to go to her parental house with my permission and has to maintain proper Purdah norms on the road” (Participant #24, age-45).

However, participant #08, a non-Muslim spouse, had non-Muslim views of the *Purdah* norm. To him, *Purdah* is a symbol of respect, in particular showing respect to older people. His reasoning demonstrates that the *Purdah* custom has evolved into a societal spectrum rather than a mere religious tenet. In his words:

“I do not think that Purdah practice is only required for Muslims. To me, Purdah is a practice that is synonymous with the veil of shame, which means not going outside and giving respect to older people. To me, Purdah means always being modest in front of elders and always bowing before them. Practicing the veil, covering the body with clothes and showing respect to them means practicing Purdah properly” (Participant#8).

Even though *Purdah* is often viewed as a religiously enforced requirement, it is worth pointing out that after these insights from Hindu participants, the concept of *Purdah* seems to extend beyond Islamic doctrinal thought and opens into rural cultural processes. For them, *Purdah* was more about showing modesty and respect toward their elders than strictly following religious rituals; thus, it proves that in rural Bangladeshi settings, *Purdah*-like practices operate as cultural norms rather than that of a strictly religious kind, and thereby it has become a tradition for all community members after being practiced for centuries, regardless of faith. Women adhere to this custom to maintain respectability in social settings.

2 ***Purdah* norms, microfinance participation, and debt burden for women**

While this second theme highlights debt burdens, a key issue is the *Purdah* culture shaping women’s access to microfinance. Respondents noted that limited movement prevented women from approaching microfinance institutions alone. Often, male family members—husband, father, or son—accompanied or handled paperwork, limiting women’s autonomy. Access was thus conditioned by social conventions, restricting their free movement and independence from the start.

**Fig 2:**
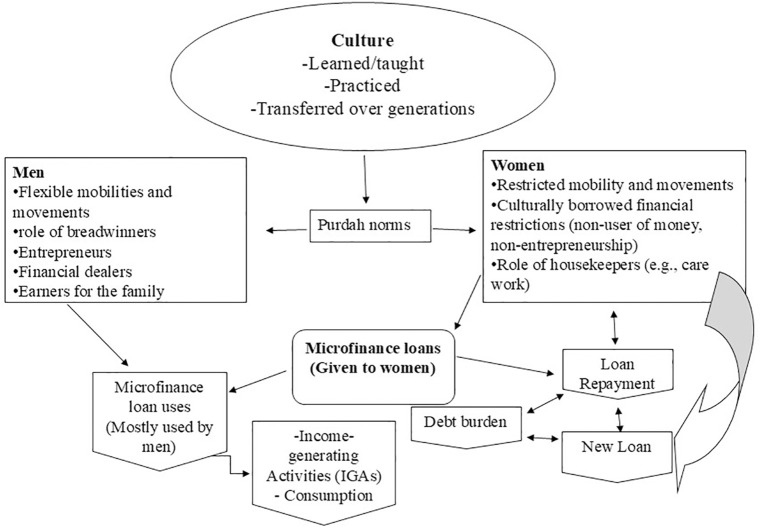
*Purdah* norm and its influence on microfinance loans. This fig illustrates that purdah norms are culturally learned, practiced, and passed down through generations, which subjects women to restrictions on mobility and financial control, while men face the opposite situation. Due to their lack of financial control and mobility, women’s microfinance loans were transferred to men. Men utilize the funds for income generation or consumption. However, loan repayment remains a legal obligation for women to MFIs, which can potentially worsen women’s debt burden if men fail to properly repay the loan installments associated with women’s loans.

The second theme of this paper comes from our field data (see Fig 2- used in this research was created by the authors of this paper specifically for this study. There do not appear to be any prior copyright issues concerning the Figure’s use for open-access publication.); it relates to the way that participants from our study learn, carry, and transfer their cultural practices, for instance, men and women idealizing *Purdah* norms due to the segregation of gendered practices. Women internalize and practice restricted mobility as a norm of *Purdah*, which prevents them from participating in economic activities such as investing in the production process, earning behavior, establishing entrepreneurship, etc. Moreover, women’s role as housekeepers limits their financial possibilities beyond the home as they are bound to carry out household duties such as cooking, cleaning, and care work. In contrast, men’s unbound freedom from *Purdah* restrictions facilitates them with flexible mobility and movement, which is a significant advantage for engaging in financial activities. As a result, men have the unique role of being the breadwinners for their families, becoming entrepreneurs, financial dealers, and economic dealers in the eyes of the community. These roles help men to maintain control over the microfinance loans, that women bring into the home from MFIs. As the data from the field shows, most participants (N = 14) reported that men had a hold on microfinance loan control and use. Men were found to be using microfinance loans for various IGAs such as fish farming, establishing small-scale businesses, and livestock farming. Moreover, men also used the loans for consumption purposes such as repairing the house and purchasing food.

In contrast, women take out loans but struggle to repay them if men fail to hand over the repayment instalments on time. In most cases, men use the money, while women rely on men’s repayments to settle the loan they borrowed from the MFIs. However, there is evidence that, in many cases, men fail to provide the repayments on time. The research participants (female microfinance borrowers) shared their experiences of having to take out further loans from other MFIs or sources (e.g., loan sharks) to repay their current loans and from further MFIs/sources to repay the newly borrowed loans. Eventually, this loan cycle increases their debt burden. This process endangers female microfinance borrowers, who fall into loan traps. Participant #17 stated in this regard:

*“When repayment is on our neck, sometimes, we take loans from other sources to repay the current one. Eventually, we take further loans to repay the new one. We are trapped in this process” (Participant #17, age=35).*

3 ***Purdah* and its influence on microfinance loans and beneficiaries**

In this study, economic independence is understood through the degree to which women have (a) the decision-making power to determine how a loan is used, (b) control over the loan and its repayment process, and (c) direct capacity to generate income through entrepreneurial or IGAs. On these grounds, economic independence does not consist merely of a loan in one’s name but must include the ability to exercise control over financial resources devoid of male intervention. Our result indicates that while *Purdah* acted to deprive women of almost all these forms of independence, cultural restrictions on their movement prevented them from using loans directly, limited their decision-making authority, and forced their dependence on men in carrying out income-generating activities.

The *Purdah* norm, which is culturally constructed and practiced, also substantially influences microfinance-receiving women’s economic independence and financial capabilities. From this perspective, we found three major sub-thematic areas (listed below) where *Purdah* was a direct or indirect cultural element that weakens women’s microfinance participation.

3.1 *Purdah* helps men take control of microfinance loans

Almost all our participants explained that men are responsible for monetary dealings in the family due to cultural practices against women’s free mobility. Therefore, the men in their families (e.g., husband, son, father-in-law) had the option to take control of microfinance loans (Such as when the women apply for the loans, they have to take consent from their husband or household head), while the women had barely any knowledge of the men’s monetary management. The microfinance-receiving women considered *Purdah* and men’s supremacy (for instance, women collected loan from MFI offices often accompanied by male relative) to be divinely and morally ordained. For example, participant #03 said:

*“Due to my Purdah regulation, I cannot participate in loan-based investments. I require my husband’s consent for loan use or investments. I am a Muslim woman, and I was taught to obey my husband’s rule. How can I use my husband’s money without his permission? As he does not permit me to use his money from the loan. I do not even know where my husband spends his money” (Participant #3, age 43).*

Notably, the male participants articulated the same beliefs and practices as the women. They perceived that women’s positions are to assist (e.g., through care work) men with domestic management, not to be earners. As per their cultural traits, practices, and religious belief systems, they explained that giving women access to money could weaken the family order and community harmony. They assumed that women would be disrespectful to *Purdah* in this way and believed that women taking part in IGAs would reduce the self-respect of men. Participant #8 explained:

*“The loan may benefit me or my family, not my wife personally. However, I must not allow her to leave home for any earning purposes. We reside in a harmonious neighborhood where men earn or deal with the money, and women care for the home. We do respect our Purdah norm, and our women are happy to follow this culture” (Participant #8, age 46).*

However, in religious belief systems around wealth ownership, specifically for Muslim women, there are prevailing contradictions with religious teaching. As mentioned explicitly by Chowdhury [3], “*women are oppressed through misinterpretation of Islam by Bangladeshi men and a section of little-learned religious leaders*”. From an early age, men’s favored interpretations and support for discourses conveyed by the (little-learned) religious leaders prevent women from availing themselves of greater opportunities [3]. In such a way, in Bangladesh, the perception of wealth ownership guides men to control women’s ownership (e.g., land, money, assets). Misleading community-level Islamic knowledge, such as the local belief that Islam says women must completely submit themselves to their husbands’ feet, works as a shield for men; however, the Holy Quran makes no reference to this [3].

3.2 *Purdah* as a cultural barrier to entrepreneurship and IGAs for women

We interviewed nine female participants who had some experience of engaging in income-generating (entrepreneurship) activities. However, their income-generating journeys were full of obstacles, as they explained. Traditional norms (*Purdah*) and cultural practices in society encourage women less to engage in monetary activities. Discriminatory behaviors such as harassment (e.g., sexual/social/oral/physical), dishonor, and family shame were commonly experienced by working women considered to be violating the *Purdah* norm. Participant #18 added:

*“Once, I tried to work outside and generate income. I worked on others’ land where many males were working. I faced bullying, and people stared at me strangely for working alongside men. Many times, I had to go there with my husband. It was unpleasant, and my family also wanted me to quit working outside” (Participant #18, age 60).*

Some participants who used loan money for income-generating activities experienced repercussions from family and community for disobeying the *Purdah* norm. For example, Participant #1 shared her experience:

*“With my microfinance loan, I initially tried to generate income. I invested in small-scale livestock rearing and then took the products (e.g., milk, chicken meat, eggs) to the nearest urban places for selling. Nevertheless, my husband reacted against my business for disobeying Purdah. He accused me of violating the Purdah norm and asked what benefit would come in the afterlife if I moved beyond home and worked outside. I was forced to close my business and abide by my Purdah rule” (Participant #1, age 25).*

However, most participants supported *Purdah* restriction as they considered it significant norm to ensure societal harmony. They opined that *Purdah* had been nurtured and practiced for generations to enable community wellbeing. According to the participants, disobeying *Purdah* and engaging in IGAs would hamper the peace of society. Men were responsible for earning and women were responsible for household management. The cooperation between men and women brought harmony to the family. Participant #21 said:

*“I never tried to work outside to generate income. It is usual to stay indoors with Purdah as men will make the money. I never had the desire to go outside to earn. I hand over my microfinance loan to my husband, who decides what to do. To my knowledge, other microfinance-receiving women do the same. Men are traditionally nurtured to go beyond home and deal with finance. I am not nurtured to disobey my husband’s word” (Participant #21, age 35).*

3.3 *Purdah* institutes economic dependency on men and increases women’s loan misery (debt burden)

As our abovementioned findings show, *Purdah* norms are normatively imposed, culturally inbound, taught, and practiced; moreover, they give men mobility and economic decisions, while failed repayment outcomes severely influence women’s loan behavior, such as ending up in formal debt from MFIs. The loans are sanctioned in women’s names, although men are the sole users. Hence, failure to repay instalments makes women loan victims, formally suffering from debt burdens. Sometimes, women apply for more loans to repay the previous ones. For example, Participant #08 said:

*“As I am not a money user, I am in great danger if my husband fails to repay the instalment on my behalf. As we are poor people, it happens many times. Every failure makes me confront the MFI’s loan collecting officer’s interrogation as I am the official defaulter on timely loan repayment. Moreover, I had to face the confrontation by staying at home as we (women) have lesser mobility beyond the threshold of our house” (Participant #8, age=46).*

In most cases, the loan repayment pressure forces women to collect additional loans from other MFIs to repay the previous ones. Eventually, the female microfinance borrowers end up in a debt trap, although they rarely use the money for themselves. Participant #17 stated, in this regard:

*“I had to take loans from organizations (MFIs) to repay my current loan instalments. It gradually increased the interest rate for both loans and eventually burdened me more if my husband or I failed to repay the instalments. Although I am not a loan user, I feel trapped with this process” (Participant #17, age 35).*

As our first theme articulated, there is evidence that the *Purdah* norm impacts microfinance recipients and their loan control or use. We sought to understand how the *Purdah* norm is understood and shapes the power relations regarding financial hegemony between men and women. Themes 2, 3, and 4 relate to the understanding of *Purdah* norms and how they shape the power relations between men and women regarding financial behavior and mobility beyond the home.

It must always be weighed that this theme concerns loan control and financial dependence rather than actually impinging upon women’s physical liberty of going places. While *Purdah* offered a system of constraints on a woman’s ability to contract loans, the broader issue of women’s physical mobility outside the home has been discussed separately in Theme 5.

4 **Marriage and *Purdah* norm**

The 25 participants shared their experiences with *Purdah* both before and after marriage, offering two distinct perspectives on the *Purdah* standard and marriage.

4.1 Practicing *Purdah* before marriage due to parental control

16 participants reported starting *Purdah* practice early, long before marriage. They had internalized it from a young age within the family (e.g., mothers, grandmothers) and parental doctrines. Most participants (N = 14) agreed that one should practice the *Purdah* norm before marriage. Participant #22, a middle-aged woman who had benefited from microfinance for 20 years, said that:

“I started maintaining Purdah before marriage. Because my family had already told me that as I was born as a girl, I must follow the veil. My father was a respected schoolteacher. As a girl, it was my responsibility to follow his every word” (Participant #22, age50).

According to two of our participants, their major motivation for practicing *Purdah* before marriage came from female neighbors. Another five participants said they felt no compulsion and were not compelled to adhere to the *Purdah* norm by their families. However, they had done so anyway, influenced by their surroundings. For example, participant #4 stated that:

“I had started following Purdah practice long before my marriage. When I saw others with veils in my childhood, I wanted to follow them, too. My parents also taught me to practice Purdah. I remember when I was a kid, I followed it by watching others. Although sometimes my parents were not that rigid in imposing Purdah at such a young age, I loved it as I saw elderly women doing that” (Participant # 4, age=55).

4.2 Practicing *Purdah* after marriage due to husband’s control

Nine participants said that they had started practicing *Purdah* after marriage. Most indicated that obligations had come either from their husbands or their in-laws. Some participants mentioned that it was very difficult for them to adapt to *Purdah* tradition as they had not been accustomed to the strictness. For example, participant #7, a woman who had been connected with microfinance for seven years, said that:

“I have to maintain strict Purdah practice since my marriage because people from my father-in-law’s house belong to a respective religious group in the village. In their family, every woman must obey the Purdah strictly. At first, it was very challenging for me to maintain a Khas veil, as I was not accustomed to this strict tradition at my father’s house. Nevertheless, I started adopting it at my in-laws’ house. I had no other choice but to adapt to the veil” (Participant #7, age=40).

Some participants (N = 9) said that although their in-laws stringently adhered to *Purdah*, their own families were less strict on it, especially when it came to wearing the *Khas* veil. Nine of the female participants reported being obligated by their husbands. In the community, it is agreed that husbands have the power to compel their wives. For example, participant #8, a husband whose household had been involved with microfinance for 15 years, said:

“My opinion is that my wife must maintain her Purdah since we got married. I am not comfortable with women not wearing a veil or meeting and greeting outsiders without Purdah. Before marriage, I told my wife’s family about my principles, as I came to know that her family allows women not to maintain Purdah. Henceforth, I faced many difficulties in getting her accustomed to Purdah after my marriage. Therefore, nurturing Purdah culture in girls from childhood is necessary” (Participant #8, age 46).

5 ***Purdah* and restriction beyond the home**

This theme examines women’s physical restrictions beyond the household. While theme 3 discussed men controlling loans through *Purdah* norms, this current theme highlights real-life barriers to women’s movement in public spaces, such as outside the home, visiting relatives, schools, markets, or workplaces. By separating financial control from mobility, we analyze each sphere and how Purdah governs both. Three sub-themes emerged.

5.1 Husbands dictating women’s mobility and freedom

Nineteen (N = 19) female participants reported that their spouses discouraged them from leaving the house. In most cases, it is the husband’s authority to enforce strict *Purdah* rules. Two participants said their husbands dislike women traveling alone, even to their parents’ home. They rarely go outside without their husbands. Participant #11 mentioned that her daughter had a bad experience at school during an exam when the teacher did not hand out the question paper on time because the daughter was reluctant to show her face in the exam hall. She explained that her daughter was only following her father’s command. Participant #1, a young woman with seven years of microfinance experience, said that:

“Yes, I think Purdah is restricting my mobility beyond the home. Because of this Purdah, my husband does not allow me to go to my father’s house. Even now, I cannot properly contact any member of my own parental house. My father was ill a few days ago, but I did not get permission to visit him. Was it my fault to be born as a girl” (Participant number #1, age 25)?

The study findings show that eight female participants had worked outside the house for financial or other purposes: their husbands and the community strongly questioning their involvement beyond household boundaries. Three participants said that few years ago they worked outside due to their financial crisis. However, their spouses and neighbors looked down on them for venturing out of the house. Female participants reported that in certain situations, their husbands were unhappy with them as they disobeyed the *Purdah* rule to leave the home. Participant #17 related the following:

“Due to Purdah practice, my mobility beyond home is forbidden. When my family needed support, I started working at other people’s houses, which caused tension between me and my husband. Moreover, the neighbors also spoke negatively about my going out. For women, the community does not accept mobility outside the home well. My husband also tortured me physically because of this. He questioned my religious sentiments. He asked me what benefit my work would bring to the afterlife if I disobeyed Purdah and worked outside” (Participant #17, age 35).

More than half (N = 13) of the participants were reluctant to go outside for work. They believed in maintaining tradition and stated that they were happy with their gender roles. For example, #16, a woman who had been connected with microfinance for five years, argued that:

“No, I do not think Purdah makes it difficult for us to go out because we do not need to go out that much. I wish not to go out because I have seen my mother doing housework since childhood, and she never required me to go outside. Although my husband does not forbid me to go beyond home, I do not feel the outside sphere is for us women [laughter]” (Participant #16, age 22).

5.2 Restriction imposed by in-laws

The study findings expound how in-laws perceive and practice the *Purdah* norm around women, particularly through the implementation of patriarchal standards and adherence to contemporary Islamic practices. In-laws create an atmosphere in which women are encouraged to remain at home in order to maintain *Purdah*, with their primary responsibility being taking care of their in-laws. These behaviors diminish women’s ability to act independently via patriarchal norms that are formed by the power dynamics between mothers-in-law and daughters-in-law, limitations on women’s bodily freedom, and women’s reliance on their male relatives [65].

Many (N = 11) participants said family members, including husbands and in-laws, were unsupportive. They faced domestic violence, like yelling, when they went outside or worked. Six (N = 6) participants were found living separately to escape violence at the in-laws’ house. For example, participant #18, a woman involved in microfinance for 22 years, said:

“No, I do not get support from my in-laws when I want to challenge the mobility-related restrictions. Moreover, my in-laws were not gracious; their manners were very authoritative towards me, so I decided to live separately from them.” (Participant #18, age 60).

A small number of participants (N = 6) reported experiencing the consequences for not adhering to *Purdah* rules or for opposing the authority of their in-laws. Participant #22 explained that her in-laws set up a second marriage for her husband and she had no say on it. She stated:

“I did not get support for my mobility at my in-laws’ house when I wanted to challenge the restrictions or faced domestic violence. Instead, they remarried my husband as a punishment for disobeying their verdict. I was mentally depressed at that time. However, they always taunt me to leave their son permanently, but I had no place to go other than this unhealthy environment” (Participant # 22, age 50).

5.3 Lack of education of men enforcing *Purdah* practice

This study shows that men’s lack of education often hinders women’s progress. Cultural practice *Purdah* reinforces gender roles and subjugates women. One participant described her husband’s disapproval of her working outside and of her breaching *Khas Purdah*, which prevented her from pursuing a government job. Female participants believe family conservatism stems from men’s lack of education, which makes spouses less sympathetic. They understand education could help, as *Khas Purdah* discourages women from formal schooling. Participant #13, a woman with ten years in microfinance, said:

“I do not support the Khas Purdah norm. I cannot entirely agree that women must cover their faces to follow the Purdah norm, called ‘Khas Purdah’. My husband is uneducated and understands nothing but to follow Khas Purdah” (Participant #13, age 45).

Participant #12 (a woman who had seven years of involvement with microfinance) explained that the lack of education among men meant that their wives had fewer employment opportunities. She believed that only women with educated husbands could thrive; she also felt that marriage was necessary for women to access financial resources. She went on to say that many women in her position were prevented from providing education for their children due to their husbands’ traditional views on the role of women in society. She said:

“Yes, Purdah is hindering my self-development. My daughter cannot go to school properly because of my husband. He did not want to invest money in my daughter’s education. My husband is illiterate and unable to understand the value of education today” (Participant #12, age 30).

## Discussion

The current study’s findings have been articulated by thoroughly explaining the connections between the *Purdah* standard and women’s microfinance. The primary aim of this study was to investigate the impact of *Purdah* norms on the economic independence of women who have received microfinance loans in Bangladesh. The study also sought to understand whether the *Purdah* norm influences microfinance loans, such as use, control and repayment, based on female borrowers’ mobility practice. We also aimed to understand *Purdah’s* perception, cultural value, and implications in everyday life. The novelty of this research is its contribution to women’s empowerment, entrepreneurship, and microfinance literature by looking at the *Purdah* norm, a concept of culture, to understand women’s financial behavior in Bangladesh. This research is limited to 25 active microfinance beneficiaries (including both women borrowers and their spouses) who had been involved in the industry for at least five years and were closely connected to *Purdah* practice. To get to the heart of the matter, a thematic analysis method is employed. Two key findings emerge: understanding the *Purdah* norm, which limits women’s mobility while granting men greater freedom, and how its cultural roots enable men to control women’s loans, thereby reducing women’s economic opportunities, as shown by MFIs.

The first finding of the research is that the *Purdah* practice is supported not just by religious beliefs but also by strict cultural norms. Most participants comprehended the *Purdah* as the veil of shame, which means not only concealing one’s body with a veil but also limiting one’s interaction with men and strangers, lowering one’s voice while speaking or laughing, and demonstrating due respect to one’s elders. The current study has also found that most rural women are limited in their ability to engage in outside employment due to their belief that doing so would breach the *Purdah* rule.

Most rural residents gave a theological interpretation of *Purdah* and utilized a religious perspective to explain it, yet they spoke of it as a cultural norm in their day-to-day lives. Men are authorized to exercise control over women’s lives, including their mobility, due to patriarchy. In their mind, the *Purdah* rule gives them the power to restrict women’s actions. Similar findings have been made elsewhere; for instance, the practice of *Purdah* is also widespread in the north and north-western regions of India, where women are excluded from both their own homes and public settings due to wearing veils, which limits their physical mobility, decision-making authority, and access to financial resources [38]. The findings of this study also echo the findings by researchers such as Cain et al. [2], Amin [1], Evertsen [66], Hossain & Hossain [67], and Lawrence & Hensly [32], who studied the influence of *Purdah* on women’s physical mobility, decision-making authority, and access to financial resources. Moreover, the practice of *Purdah is* found to be uniform both before (parental influence) and after (husband/in-laws influence) marriage in this research and studies from Chan *et al.*, [68], Jennings *et al*., [35] and Kabeer [69]. This current study has identified a gap in previous research [70–74] where researchers argued that women’s misery caused by lack of mobility grows intense after marriage and that women’s lack of education makes the situation worse.

Literature has also articulated gender-based educational disparity and its effects on women’s empowerment due to their illiteracy and lack of awareness and knowledge [71,73,74]. However, the previous research has provided little insight into the structural barriers restricting women’s mobility, such as early childhood learning and socialization. In this area, our study provides a supplementary result; it is impossible to empower women without the proper assistance of men, which could be made possible by creating awareness through education. By providing equal opportunity for boys and girls within the family to question *Purdah* limitations, these findings open the door to understanding the importance of correct socialization from childhood. The findings highlight the common understanding of what it means for *Purdah* standards to limit women’s mobility.

This research’s second finding showed that women’s lack of mobility, which is restricted by *Purdah*, left them with minimal chances to be involved in financial activities such as investment, entrepreneurship, or economic dealings. Instead, more freedom for men in public spaces helped them take control over women’s microfinance loans. From this perspective, this research developed a framework (see Fig 2) explaining how *Purdah* in Bangladesh gives men more flexibility and economic freedom while enforcing entrepreneurial restrictions for women. Our data shows that culture is the foundational structure for regulating men’s and women’s gender roles. In Bangladesh, *Purdah* separates women from men by imposing restrictions on mobility beyond the home boundary, assigning housekeeping roles, and discouraging women from earning money for their families. As a result, although microfinance loans are designed for women to be included in financial activities (e.g., IGAs, entrepreneurship), men are the sole users of the loan money as they are dictated by culture to carry the role of earners. However, the legality of the loan repayment remains a formal responsibility for women. In many cases, women have to take further loans from other MFIs, which they are required to repay in the same way as the previous loan. Failure to repay previous or newly taken loans create a debt burden for women, who find the process to be a loan trap for themselves.

This final finding of our study corroborates the findings of Kabeer [75], Karim [24,58], Davis [76], and Shohel *et al*. [4,5] that men use female-driven (microfinance loans) money. However, the originality of this study is that it has attempted to explain a cultural norm, i.e., *Purdah*, and its role, while showing that the supposed ability of microfinance loans to serve and benefit women is not truly the case. Studying at *Purdah* as a culturally driven normative force that controls microfinance-receiving women’s mobility and economic participation is this study’s unique contribution to microfinance, women’s empowerment, and entrepreneurship literature.

### Implications for future researchers

The current study’s findings may be useful for academics interested in examining the effects of the *Purdah* norm on women’s subordination in rural areas. Despite the study’s focus on the southern part of Bangladesh, it may inspire researchers to look at *Purdah*, mobility restrictions, and women’s subordination in other regions of the country or across nations. While this study has extensively explored the topic within its scope, the researchers still recommend further research for those seeking a deeper understanding. More explicitly, there should be further examination of the gender role construction process (e.g., by socialization) and male educational ability to challenge and scrutinize conventional gender norms.

### Limitations and challenges of the research

As this study relies on qualitative analysis, its findings should not be extrapolated to the whole of Bangladesh. Due to patriarchal constraints and traditional attitudes, acquiring sufficient information from the participants was difficult, especially since the current study’s major goal was connected to the *Purdah* practice. Challenges in efficiently carrying out the research included a lack of time and resources. The study’s findings, however, provide light on the extent to which *Purdah* rules limit women’s mobility and the variables that play a role in this restriction. Another challenge the authors acknowledge is the *Emic* (insiders’ viewpoint) and *Etic* (outsiders’ viewpoint) perspectives of the data acquired from this qualitative research. This challenge has arisen as the data collected from the participants was subjective in nature. To overcome the challenge, the researchers adopted a value-free perspective during the analysis, assigning concepts and themes to the subjects under study. Moreover, when further investigating this study’s findings, future researchers might study *Purdah* and its practices and implications by comparing local traditions, beliefs, or practices with Islamic doctrine and teaching perspectives. Any discrepancy between local practices and Islamic doctrine-based teaching would significantly contribute to further literature.

## Conclusion

This research has aimed to explain the *Purdah* norm, its construction process, and the way its practices affect the economic domain of female microfinance clients in Bangladesh. *Purdah* norms have been found to limit women’s independence in rural Bangladesh. The research shows that cultural norms (e.g., beliefs and practices) prevent women from engaging in economic pursuits. People’s minds are also rendered more conventional by gender indoctrination, which benefits men disproportionately. In this study, some rural women were discovered to be actively engaging in IGAs in order to break out of their subordinate status. Of those who did, the vast majority were affected by patriarchal norms, religious superstitions, and cultural subordination. The *Purdah* standard has also been found to be a tool of male domination, such as controlling and using the microfinance loans disbursed to women. Through snowball sampling, this study has investigated the larger population of rural Bangladeshi microfinance recipients, their views on *Purdah*, the restrictions it imposes on women’s mobility, and the community’s religious and cultural attitude towards women working outside the home. The research required input from all women who have received microfinance in Bangladesh, which is unrealistic; therefore, it may have failed to provide absolute clarity on the concerns. However, policymakers may benefit from the study’s findings by learning more about the effects of *Purdah* on women who receive microloans, as well as the challenges these women face and how they might be addressed, such as promoting gender equality in education and socialization, eliminating harmful religious beliefs and superstitions, and limiting male dominance in society.

## Recommendations

Based on the findings from the field, this study has some recommendations that would help to increase women’s mobility and the effectiveness of development initiatives taken for women’s empowerment, such as microfinance. The recommendations are,

- Microfinance institutions should introduce recurring training and awareness-building sessions for both men and women regarding women’s empowerment issues such as mobility, income, and household decision-making.
- Changes in gender norms impacting the effectiveness of micro-finance initiatives, particularly governmental initiatives, are required.
- Effective teaching/learning programs for female student socialization, may be designed and added to microfinance operations and delivered as a package for the stakeholders where gender experts, academics and expert researchers could play a role to minimize gender-based disparities in agency and practice.

## Supporting information

S1 Map Study Location. (TIF)

S1 File Data availability. (DOCX)
