# Supplementary figures and images for: “Stay indoors with Purdah, men will make the money”: A qualitative study investigating women’s microfinance participation and mobility practices in Bangladesh
Source: PLoS One. 2026 Apr 2;21(4):e0346323. doi: 10.1371/journal.pone.0346323 (PMC13046111; doi:10.1371/journal.pone.0346323)

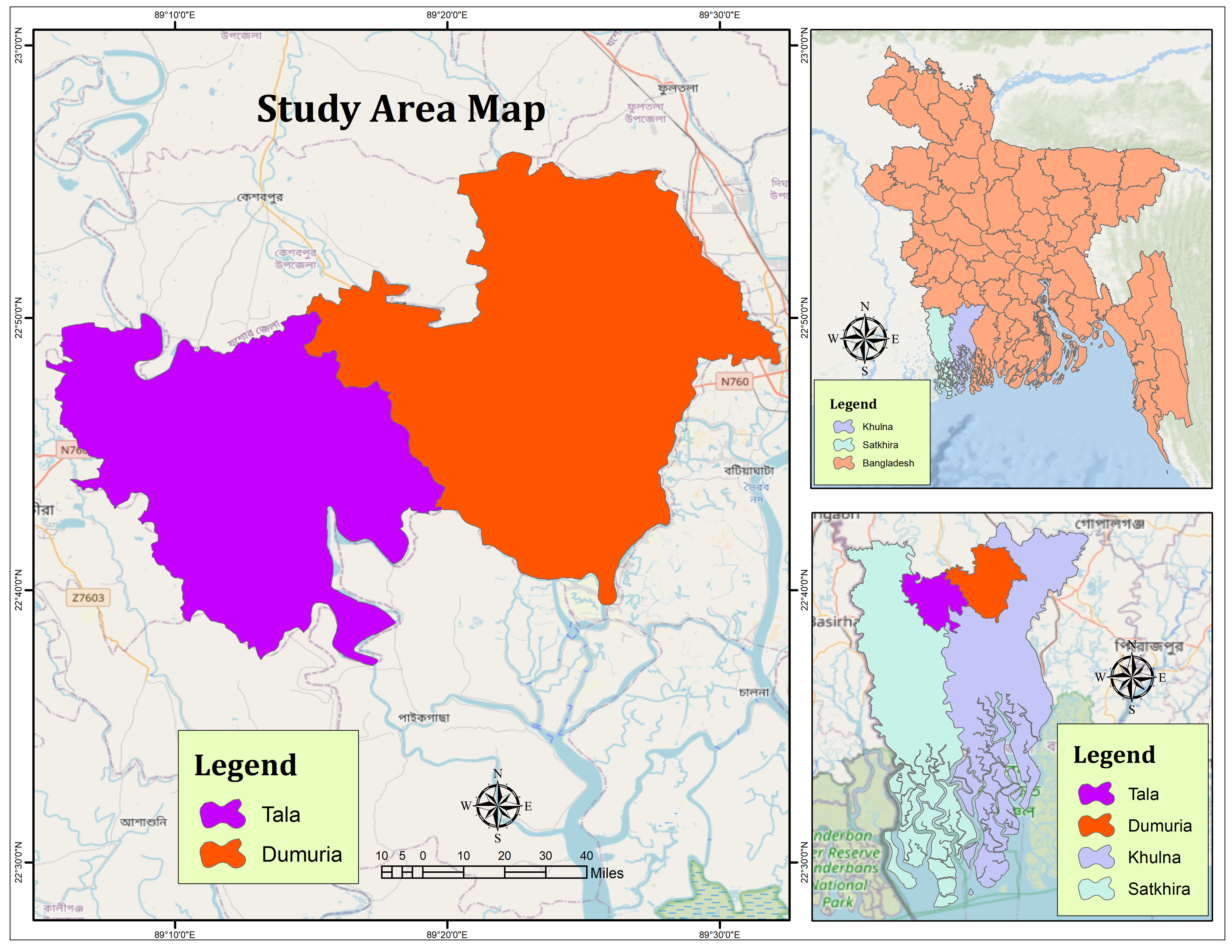

Supplement: S1 Map — (TIF) [file pone.0346323.s001.tif]
