# Supplementary material for: “Stay indoors with Purdah, men will make the money”: A qualitative study investigating women’s microfinance participation and mobility practices in Bangladesh
Source: PLoS One. 2026 Apr 2;21(4):e0346323. doi: 10.1371/journal.pone.0346323 (PMC13046111; doi:10.1371/journal.pone.0346323)
Supplement: S1 File — (DOCX) [file pone.0346323.s002.docx]

All data used in the article are available in this file,

“By Purdah, I mean the order of Allah and the lifestyle shown by our Prophet (SM). If I maintain Purdah properly, people will appreciate me, and it will protect me from “Kunazar”.^^[[1]](#footnote-1)^^My husband will also be happy. As a woman, I must make my husband happy. If I do it properly, Allah will be happy with my deeds” (Participant #15, age 25).

“By Purdah, I mean that Muslim women must follow it. Mother Fatima used to follow Purdah. It is the order of our last Prophet from Allah. However, I also think it is possible to satisfy Allah by following prayer, being honest, and practicing Khas Purdah. Practicing Khas Purdah without being honest and religious is useless” (Participant #14, age 30).

“By ‘Purdah’, I mean that you cannot go in front of a man; you have to speak in a lower voice, stay indoors even within home, obey your husband’s order, and pray five times a day” (Participant # 23, age 30).

“By the concept of Purdah, I understand [it as] not only staying inside the home but also covering my face in front of my husband when we meet or greet, being polite with him and obeying his will” (Participant #17, age 35).

“I understand, Purdah, that as a woman, she must not show her face to outsiders. If required, she must not go in front of other men without my permission and without covering her face. She is only allowed to go to her parental house with my permission and has to maintain proper Purdah norms on the road” (Participant #24, age-45).

“I do not think that Purdah practice is only required for Muslims. To me, Purdah is a practice that is synonymous with the veil of shame, which means not going outside and giving respect to older people. To me, Purdah means always being modest in front of elders and always bowing before them. Practicing the veil, covering the body with clothes and showing respect to them means practicing Purdah properly” (Participant#8).

*“When repayment is on our neck, sometimes, we take loans from other sources to repay the current one. Eventually, we take further loans to repay the new one. We are trapped in this process” (Participant #17, age=35).*

*“Due to my Purdah regulation, I cannot participate in loan-based investments. I require my husband’s consent for loan use or investments. I am a Muslim woman, and I was taught to obey my husband’s rule. How can I use my husband’s money without his permission? As he does not permit me to use his money from the loan. I do not even know where my husband spends his money” (Participant #3, age 43).*

*“The loan may benefit me or my family, not my wife personally. However, I must not allow her to move beyond home for earning purposes. We reside in a harmonious neighborhood where men earn or deal with the money, and women care for the home. We do respect our Purdah norm, and our women are happy to follow this culture” (Participant #8, age 46).*

*“Once, I tried to work outside and generate income. I worked on others’ land where many males were working. I faced bullying, and people stared at me strangely for working alongside men. Many times, I had to go there with my husband. It was unpleasant, and my family also wanted me to quit working outside” (Participant #18, age 60).*

*“With my microfinance loan, I initially tried to generate income. I invested in small-scale livestock rearing and then took the products (e.g., milk, chicken meat, eggs) to the nearest urban places for selling. Nevertheless, my husband reacted against my business for disobeying Purdah. He accused me of violating the Purdah norm and asked what benefit would come in the afterlife if I moved beyond home and worked outside. I was forced to close my business and abide by my Purdah rule” (Participant #1, age 25).*

*“I never tried to work outside to generate income. It is usual to stay indoors with Purdah as men will make the money. I never had the desire to go outside to earn. I hand over my microfinance loan to my husband, who decides what to do. To my knowledge, other microfinance-receiving women do the same. Men are traditionally nurtured to go beyond home and deal with finance. I am not nurtured to disobey my husband’s word” (Participant #21, age 35).*

*“As I am not a money user, I am in great danger if my husband fails to repay the instalment on my behalf. As we are poor people, it happens many times. Every failure makes me confront the MFI’s loan collecting officer’s interrogation as I am the official defaulter on timely loan repayment. Moreover, I had to face the confrontation by staying at home as we (women) have lesser mobility beyond the threshold of our house” (Participant #8, age=46).*

*“I had to take loans from organizations (MFIs) to repay my current loan instalments. It gradually increased the interest rate for both loans and eventually burdened me more if my husband or I failed to repay the instalments. Although I am not a loan user, I feel trapped with this process” (Participant #17, age 35).*

“I started maintaining Purdah before marriage. Because my family had already told me that as I was born as a girl, I must follow the veil. My father was a respected schoolteacher. As a girl, it was my responsibility to follow his every word” (Participant #22, age50).

“I had started following Purdah practice long before my marriage. When I saw others with veils in my childhood, I wanted to follow them, too. My parents also taught me to practice Purdah. I remember when I was a kid, I followed it by watching others. Although sometimes my parents were not that rigid in imposing Purdah at such a young age, I loved it as I saw elderly women doing that” (Participant # 4, age=55).

“I have to maintain strict Purdah practice since my marriage because people from my father-in-law’s house belong to a respective religious group in the village. In their family, every woman must obey the Purdah strictly. At first, it was very challenging for me to maintain a Khas veil, as I was not accustomed to this strict tradition at my father’s house. Nevertheless, I started adopting it at my in-laws’ house. I had no other choice but to adapt to the veil” (Participant #7, age=40).

“My opinion is that my wife must maintain her Purdah since we got married. I am not comfortable with women not wearing a veil or meeting and greeting outsiders without Purdah. Before marriage, I told my wife’s family about my principles, as I came to know that her family allows women not to maintain Purdah. Henceforth, I faced many difficulties in getting her accustomed to Purdah after my marriage. Therefore, nurturing Purdah culture in girls from childhood is necessary” (Participant #8, age 46).

“Yes, I think Purdah is restricting my mobility beyond the home. Because of this Purdah, my husband does not allow me to go to my father’s house. Even now, I cannot properly contact any member of my own parental house. My father was ill a few days ago, but I did not get permission to visit him. Was it my fault to be born as a girl” (Participant number #1, age 25)?

“Due to Purdah practice, my mobility beyond home is forbidden. When my family needed support, I started working at other people’s houses, which caused tension between me and my husband. Moreover, the neighbors also spoke negatively about my going out. For women, the community does not accept mobility outside the home well. My husband also tortured me physically because of this. He questioned my religious sentiments. He asked me what benefit my work would bring to the afterlife if I disobeyed Purdah and worked outside” (Participant #17, age 35).

“No, I do not think Purdah makes it difficult for us to go out because we do not need to go out that much. I wish not to go out because I have seen my mother doing housework since childhood, and she never required me to go outside. Although my husband does not forbid me to go beyond home, I do not feel the outside sphere is for us women [laughter]” (Participant #16, age 22).

“No, I do not get support from my in-laws when I want to challenge the mobility-related restrictions. Moreover, my in-laws were not gracious; their manners were very authoritative towards me, so I choose to live separately from them.” (Participant #18, age 60).

“I did not get support for my mobility at my in-laws’ house when I wanted to challenge the restrictions, ofaced domestic violence. Instead, they remarried my husband as a punishment for disobeying their verdict. I was mentally depressed at that time. However, they always taunt me to leave their son permanently, but I had no place to go other than this unhealthy environment” (Participant # 22, age 50).

“I do not support the Khas Purdah norm. I cannot entirely agree that women must cover their faces to follow the Purdah norm, called ‘Khas Purdah’. My husband is uneducated and understands nothing but to follow Khas Purdah” (Participant #13, age 45).

“Yes, Purdah is hindering my self-development. My daughter cannot go to school properly because of my husband. He did not want to invest money in my daughter’s education. My husband is illiterate and unable to understand the value of education today” (Participant #12, age 30).

1. A Bangla word that means “evil eye”. Here, this word is used to indicate that *Purdah* is a protector from the evil eye. It refers to wishing someone bad luck or illness, which is generated through jealousy. [↑](#footnote-ref-1)
